# Residual shunt in an infant following patent ductus arteriosus ligation detected via transesophageal echocardiography monitoring during pulmonary artery banding: a case report

**DOI:** 10.1186/s40981-019-0240-3

**Published:** 2019-03-08

**Authors:** Takayuki Yoshida, Natsuki Anada, Yasufumi Nakajima

**Affiliations:** Department of Anesthesiology, Kansai Medical University Hospital, 2-3-1 Shin-machi, Hirakata City, Osaka 573-1191 Japan

To the Editor,

Patent ductus arteriosus (PDA) often requires surgical intervention in neonates and infants. During PDA closure, transesophageal echocardiography (TEE) provides consecutive monitoring of ductal flow, allowing detection of a residual shunt and final confirmation of PDA closure [1–3]. However, no reports exist of a residual PDA shunt being detected during pulmonary artery banding (PAB) manipulation on intraoperative TEE monitoring. Here, we describe a low-weight infant in whom a residual shunt following PDA ligation was detected via TEE monitoring during subsequent PAB.

## Case presentation

**Fig. 1:**
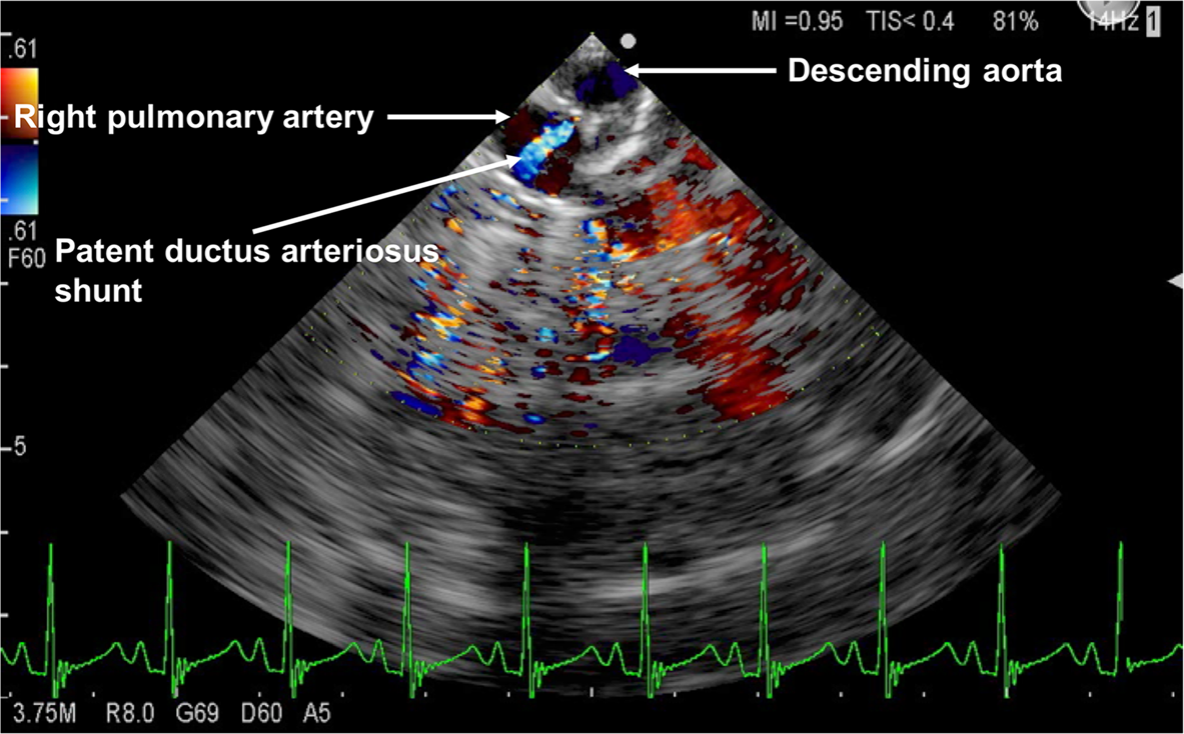
Residual PDA shunt flow on color Doppler flow imaging**.** A mosaic jet moving from the descending aorta toward the pulmonary artery, indicating a residual PDA shunt, is seen

A 6-month-old girl (2.7 kg) diagnosed with PDA, ventricular septal defect, and accompanying pulmonary hypertension was scheduled for PDA ligation and PAB. Preoperative transthoracic echocardiography indicated the peak pressure gradient of a left-to-right PDA shunt as 14 mmHg. After induction of general anesthesia and tracheal intubation, a single-plane TEE probe (UST-52110S; Hitachi-Aloka Medical, Tokyo, Japan), connected to an ultrasound apparatus (Prosound F75; Hitachi-Aloka Medical), was inserted into the patient’s esophagus. The tip of this probe was 6.0, 13.4, and 5.3 mm in width, length, and depth, respectively. Hemodynamics and ventilation conditions were unchanged following probe insertion and manipulation. Before surgery initiation, TEE demonstrated a left-to-right shunt through the PDA on color Doppler flow imaging. Surgery was initiated under midline sternotomy, and the PDA was ligated using silk thread. After PDA ligation, disappearance of the PDA shunt flow was confirmed. Subsequently, when the surgeon was adjusting the diameter of band on the main pulmonary artery during PAB, TEE monitoring revealed a residual PDA shunt on color Doppler imaging. There was a discrete mosaic jet moving from the descending aorta toward the pulmonary artery (Fig. 1), suggesting incomplete PDA ligation. The surgeon consequently applied a hemoclip to the PDA, and the shunt was confirmed to have disappeared on TEE. The main pulmonary artery was finally strangulated using a 24-mm band, resulting in a peak pressure gradient of 41.9 mmHg at the PAB site when the systemic arterial pressure was 74/45 mmHg. With no residual PDA flow thereafter, the patient successfully underwent ventricular septal defect closure and pulmonary arterioplasty at the age of 17 months.

## Discussion

To our knowledge, this is the first report describing a residual PDA shunt that was detected again with TEE during the PAB procedure after ductal flow had been confirmed to have ceased after PDA ligation. We believe that decreased pulmonary arterial pressure caused by PAB greatly increased the pressure gradient between the pulmonary artery and the descending aorta, which made the residual PDA shunt detectable in our case.

Hemodynamic compromise and/or airway obstruction can be caused by a TEE probe especially when observing blood flow around the pulmonary artery in pediatric patients [4, 5]. A thin single-plane TEE probe can reduce the risk of these complications while providing sufficient information to determine PDA shunt flow in pediatric patients, as noted in our case.

We suggest that, in surgery for PDA closure in combination with PAB, the existence of residual PDA shunt flow should be evaluated again after the pulmonary artery pressure decreases with banding.

## Abbreviations

PAB: Pulmonary artery banding
PDA: Patent ductus arteriosus
TEE: Transesophageal echocardiography
